# Supplementary material for: MAME: Multidimensional adaptive metamer exploration with human perceptual feedback
Source: J Vis. 2026 Aug 3;26(8):1. doi: 10.1167/jov.26.8.1 (PMC13440621; doi:10.1167/jov.26.8.1)
Supplement: Supplementary file 1 [file jovi-26-8-1_s001.pdf]

Supplementary Materials

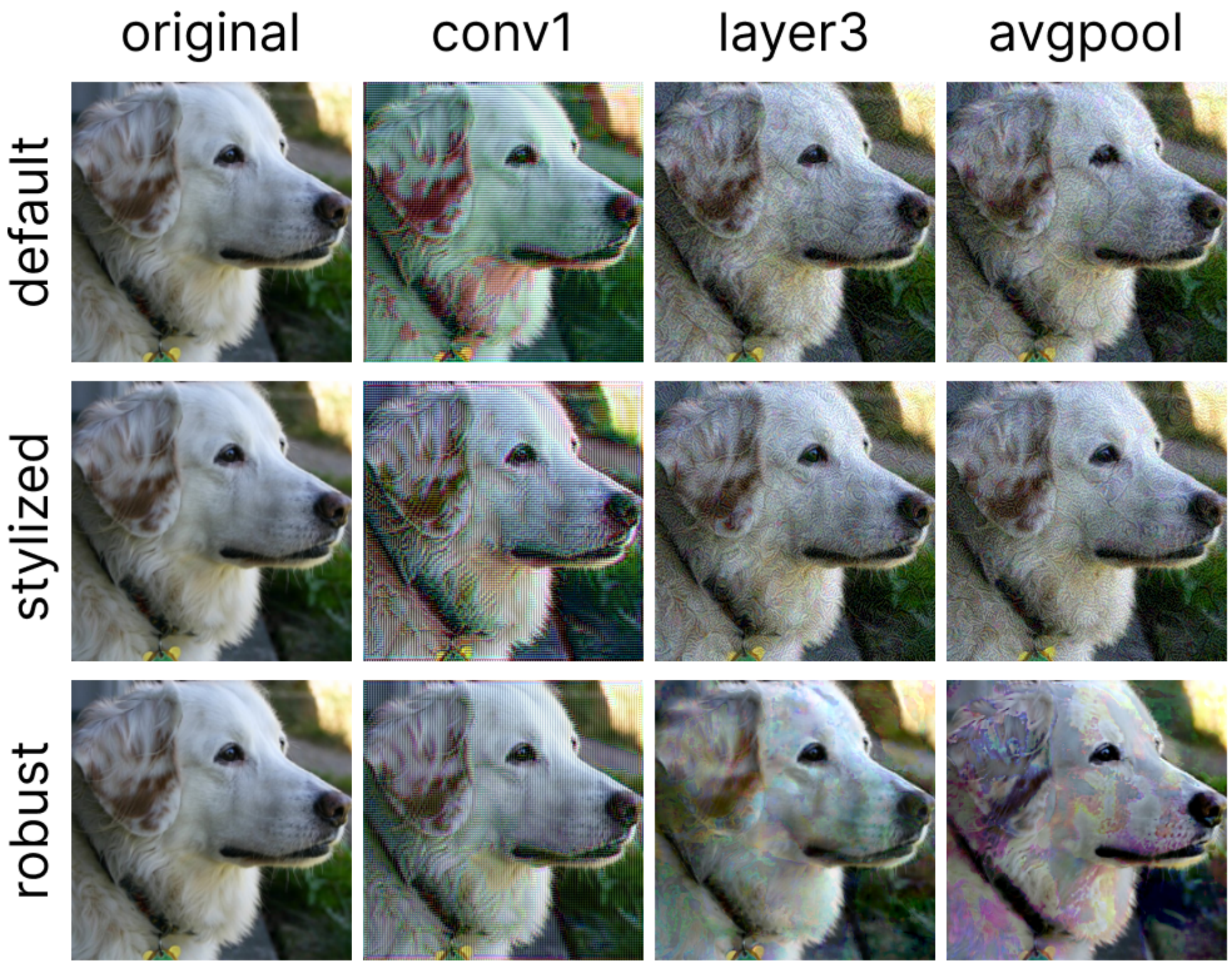

Figure S1: Examples of image modulation using models with different training regimes but the same architecture as the robust ResNet50 used in this study. The robust ResNet was trained to be resistant to adversarial attacks, whereas the default model was trained on standard ImageNet data, and the stylized model was trained on stylized versions of ImageNet (Geirhos, Rubisch, et al., 2018). When the same modulation parameters were applied to each model, we found that, particularly in higher layers, the default and stylized models failed to produce clear modulations from the original image, in contrast to the robust model (cf. Feather, 2023).

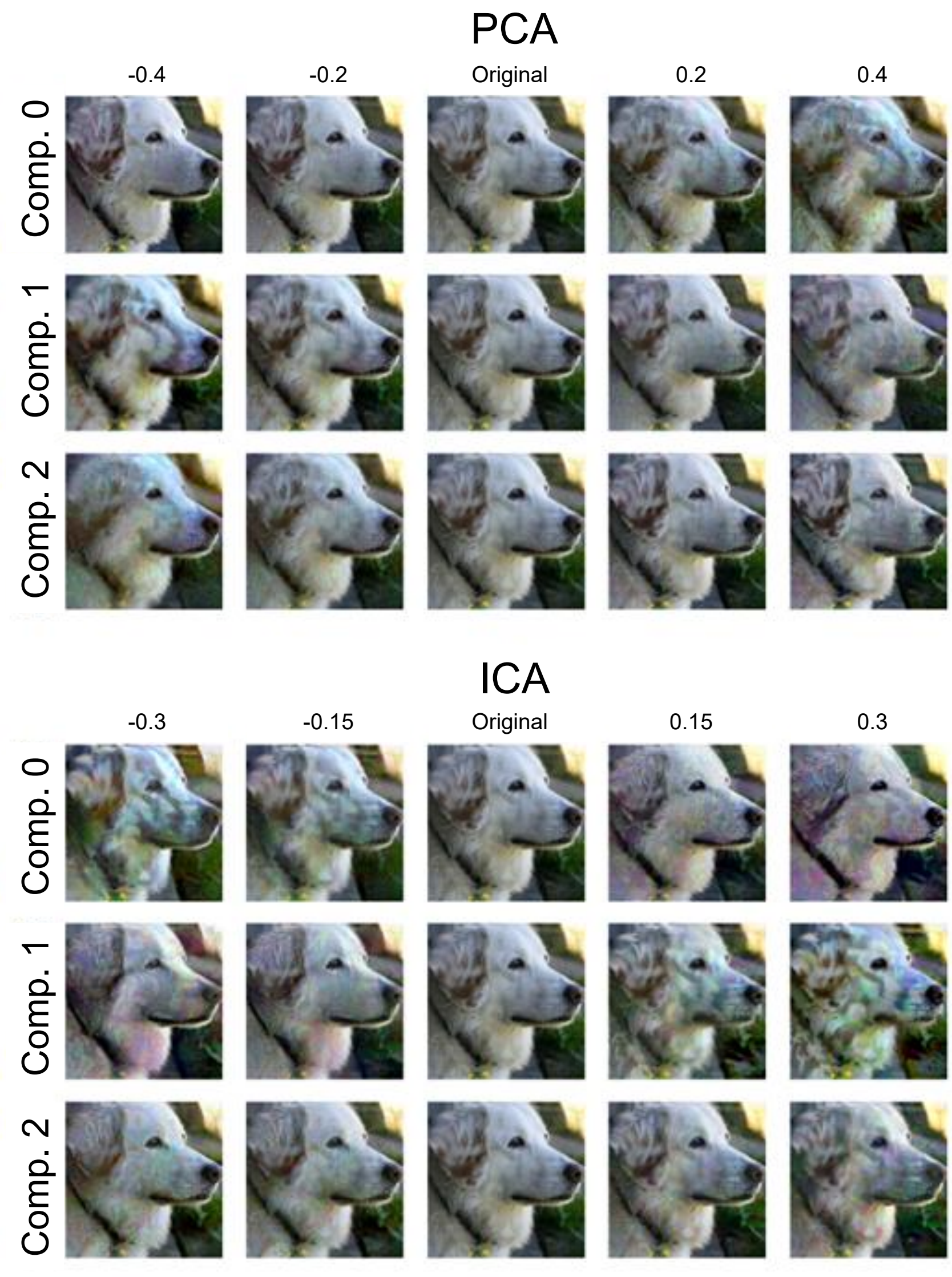

Figure S2: Comparison of image modulation using PCA and ICA in high-level representations (computed at the `avgpool` layer of the robust ResNet50). (Upper) Three principal components were extracted via PCA from vectorized Gram matrices, and image modulation was performed along each component axis. (Bottom) Image modulation using ICA, as employed in the present study. Due to the learning properties of DNNs, high-level features are expected to contain many independent components. The PCA-based modulation, which preserves orthogonality in the Gram matrix space, fails to capture such diversity, making it difficult to generate varied image modulations.

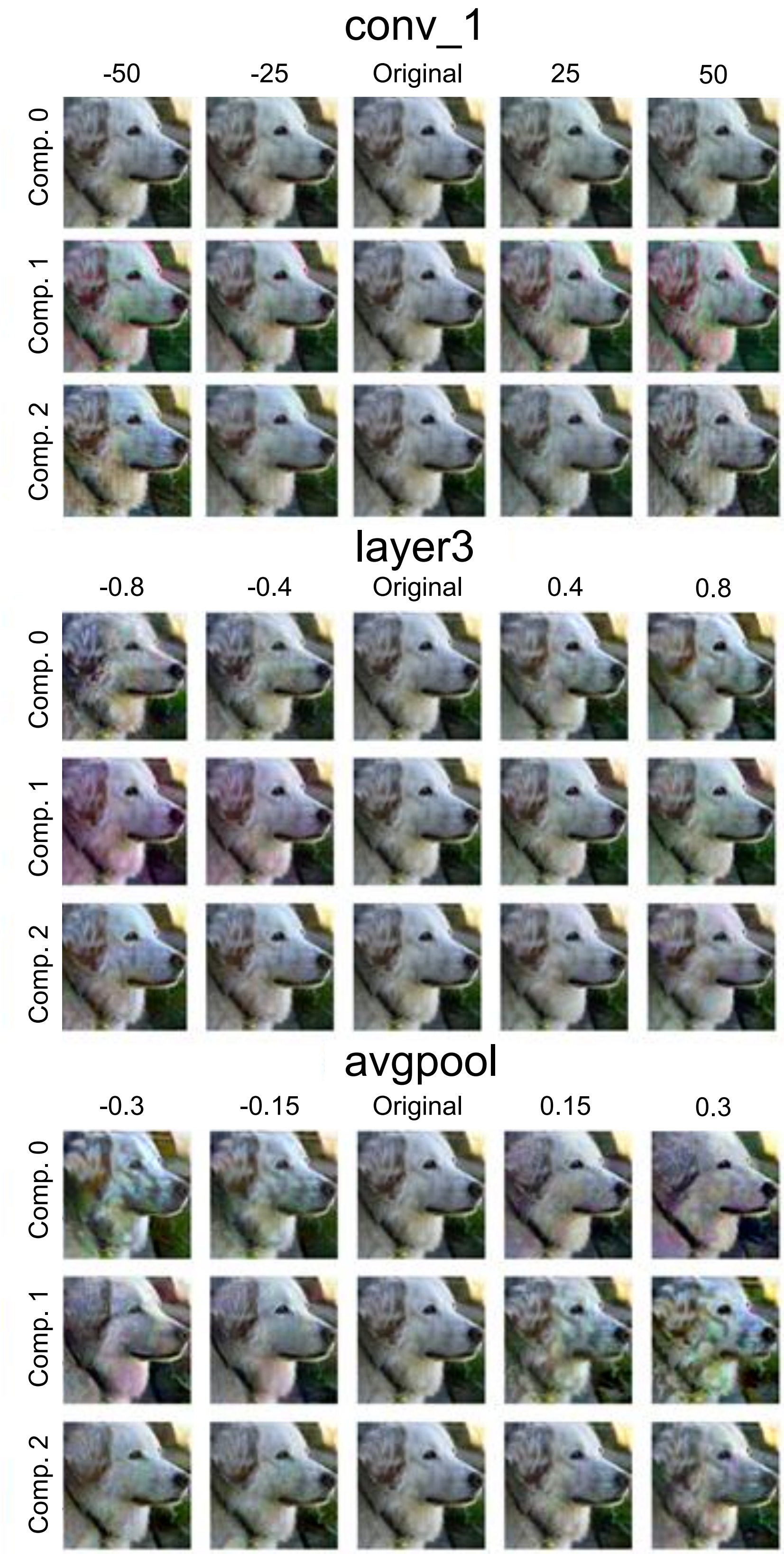

Figure S3: Examples of image modulation for each layer and each component. This figure presents a finer-step version of Fig. 4.

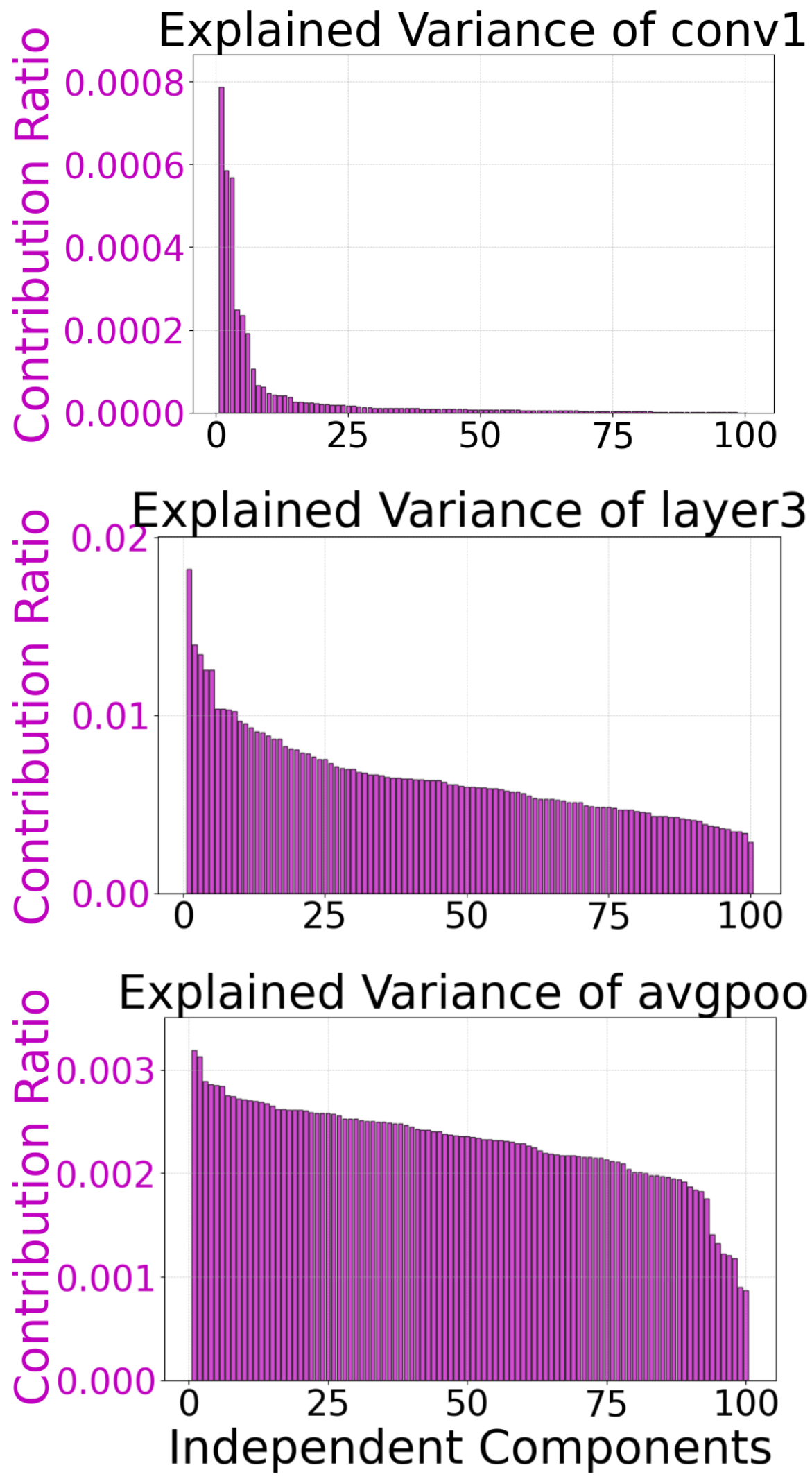

Figure S4: Explained variance of ICA components sorted in descending order. (Top) `conv1`, (Middle) `layer3`, (Bottom) `avgpool`.

Table S1: Normalized final loss for each layer, ICA component, and direction (mean ± SD, n = 100). The normalized final loss is defined as the ratio between the final loss and the initial loss before optimization. For each layer, target values were sampled uniformly in log-space (i.e., log-spaced sampling) within the specified $\log_{10}(t)$ range.

Layer	$\log_{10}(t)$ range	Component	Direction	Normalized final loss
`conv1`	[0.0, 3.0]	0	+	0.006636 ± 0.024610
			−	0.007039 ± 0.027097
		1	+	0.004328 ± 0.010553
			−	0.004179 ± 0.010673
		2	+	0.005512 ± 0.010508
			−	0.007136 ± 0.014643
`layer3`	[-2.0, 1.0]	0	+	0.006004 ± 0.012682
			−	0.005656 ± 0.010533
		1	+	0.007383 ± 0.014922
			−	0.006431 ± 0.014312
		2	+	0.005035 ± 0.008581
			−	0.006483 ± 0.011846
`avgpool`	[-2.0, 1.0]	0	+	0.013961 ± 0.055785
			−	0.011262 ± 0.044577
		1	+	0.006213 ± 0.021678
			−	0.007241 ± 0.016612
		2	+	0.008365 ± 0.026241
			−	0.006456 ± 0.017010

Table S2: Threshold estimates based on staircase reversal points. In addition to the psychometric-function analysis reported in Table 2, thresholds were estimated by averaging the last five reversal points of the staircase trajectory for each condition.

Layer	Eccentricity (°)	Mean	Std
`conv1`	4	47.915	8.8166
	8	63.0404	11.8613
	12	63.7918	8.5024
`layer3`	4	0.486	0.1275
	8	0.5983	0.0686
	12	0.6407	0.0533
`avgpool`	4	0.1538	0.0182
	8	0.153	0.0253
	12	0.1688	0.0203

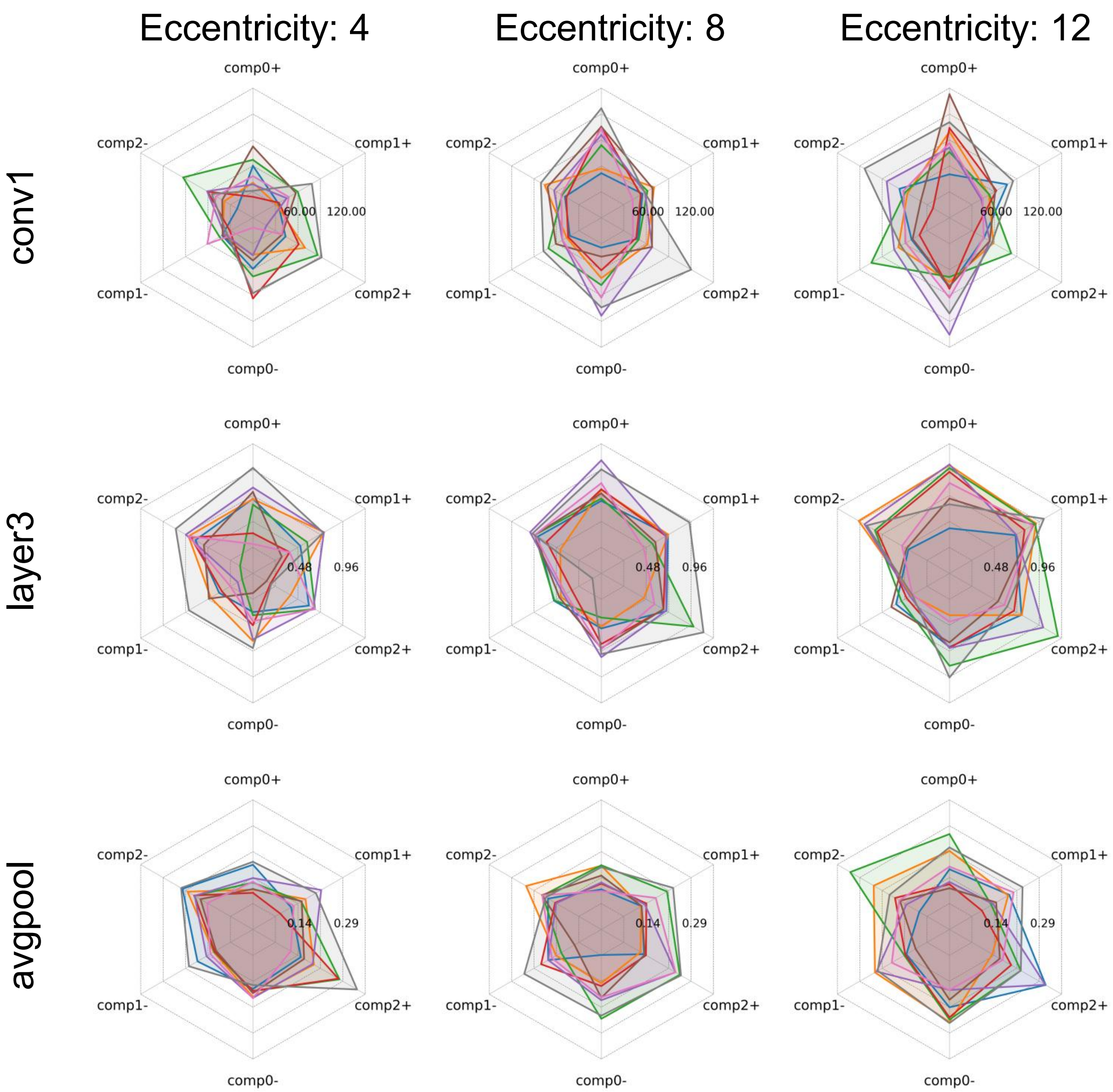

Figure S5: The ICA threshold values obtained from all subjects (n = 8) across nine radar charts. Each row corresponds to a convolutional layer, and each column represents the eccentricity (in degrees) at which the stimulus was presented. The ICA threshold value at each point indicates the mean of reversal points obtained through the staircase method ($n \geq 6$). Colors distinguish individual subjects, with the same color consistently representing the same subject across all charts.

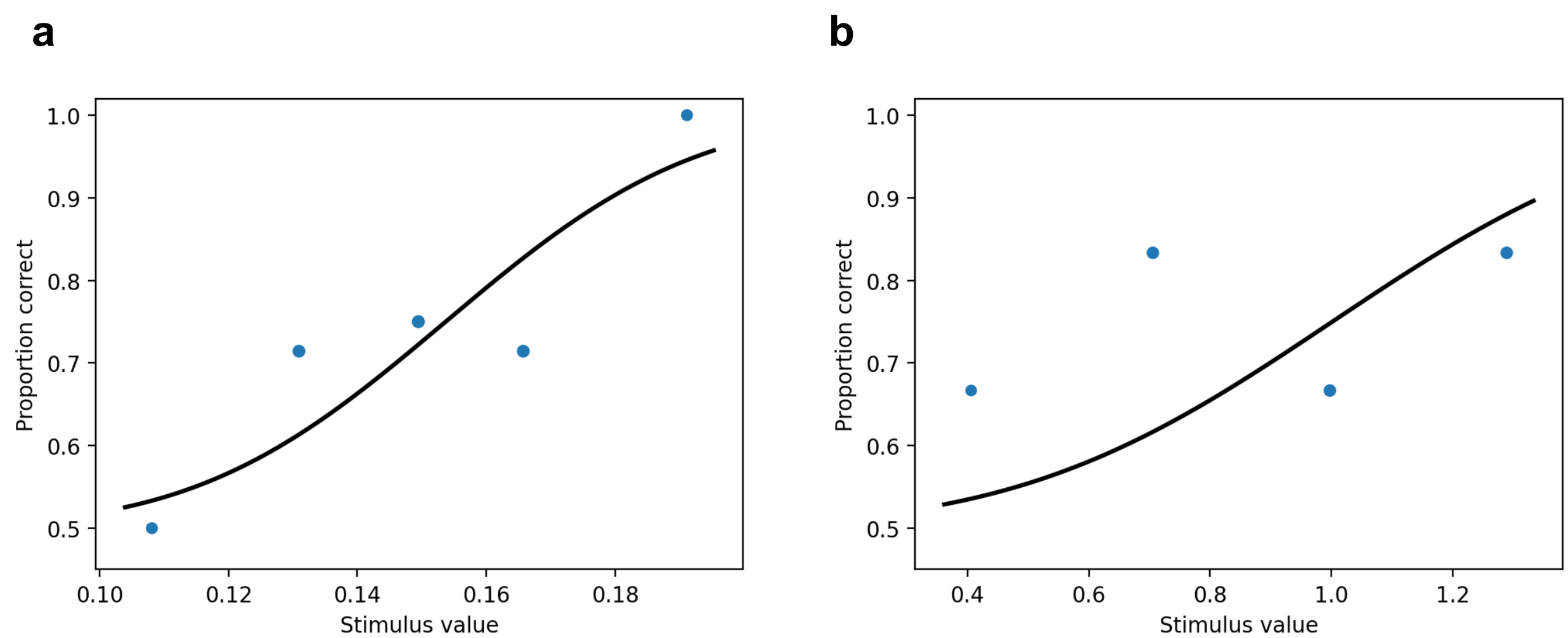

Figure S6: Example psychometric function fits from a single participant. Thresholds in the main analysis were estimated by fitting sigmoidal functions. Because some fits may not adequately capture the data (e.g., b), we additionally analyzed the data using reversal averages from the classical staircase procedure and obtained similar results (Tables S2). We also confirmed that fit quality was comparable across layer conditions (RMSE: `conv1` = 0.11, `layer3` = 0.10, `avgpool` = 0.13).

Table S3: Results of the two-way repeated-measures ANOVA for SSIM metrics. Reported p values are Greenhouse–Geisser corrected.

Source	df	F	p	η_g^2
Layer	(2, 14)	696.67	$< .001$	0.936
Eccentricity	(2, 14)	30.91	$< .001$	0.346
Layer × Eccentricity	(4, 28)	7.00	.016	0.218

Table S4: Results of the two-way repeated-measures ANOVA for LPIPS metrics. Reported p values are Greenhouse–Geisser corrected.

Source	df	F	p	η_g^2
Layer	(2, 14)	826.47	$< .001$	0.937
Eccentricity	(2, 14)	38.25	$< .001$	0.368
Layer × Eccentricity	(4, 28)	5.30	.044	0.183

Table S5: Results of the two-way repeated-measures ANOVA for DISTS metrics. Reported p values are Greenhouse–Geisser corrected.

Source	df	F	p	η_g^2
Layer	(2, 14)	1871.61	$< .001$	0.980
Eccentricity	(2, 14)	23.16	$< .001$	0.316
Layer × Eccentricity	(4, 28)	8.47	.015	0.248
